# The Germicidal Action of the Blood

**Published:** 1891-03

**Authors:** J. Wellington Byers

**Affiliations:** Charlotte, N. C.


### Selections and Abstracts

### THE GERMICIDAL ACTION OF THE BLOOD.

*To the Editor of the Medical Record:*

SIR: In view of the fact that medical science is attaching unusual signification to the discovery of the "germicidal action of the blood" I desire to direct attention to that portion of its origin and history connected with my observations made several years since. Metschnikoff and his theory of the phagocytic action of cells has attracted universal notice and has brought its distinguished propounder a deserved fame. At present there is a disposition generally prevalent which is inclined to look upon the discovery of the germicidal powers of the blood as of far greater importance than that of the noted Russian; yet as to whether it shall eventually supplant or only supplement his services remains to be seen. Be this as it may, my sole purpose here is to ascertain, if possible, to whom the credit of first suggesting this function of blood belongs. Without arrogating this distinguishing fact to myself as author, I do maintain that I advanced this doctrine, independently and originally, so far as I know, as far back as September, 1887, while preparing the MS. for the article "Race" in Wood's Handbook, vol. viii., page 430, where I employed the following language when discussing the factors which go to determine the conditions of immunity and susceptibility among the various races and nations of the world: "Whether the active mediums of resistance be chemical substances, the activities of the cellular tissues, or the white blood corpuscles, or all of these in combination, we know that the investigations of Maurel show the blood of the different races presents differences of composition and number of cells. . . . How far these discrepancies are to be taken in explanation of the various degrees of immunity and susceptibility that are found to characterize the various people of the world is a question still demanding further inquiry. Viewing 'the blood as the life' and that a capacity to resist any and all diseases of this class—infectious—is proportional to its perfectness in composition, it can readily be seen that the blood may also act the part of a protecting agency—one capable of thwarting the assaults of the pathogenic micro-organism." I base my pretentions to priority upon the date of September, 1887, when, so far as I am aware, no author had suggested or discovered the germicidal action of the blood. If I am wrong I should be happy to be corrected. Respectfully, J. WELLINGTON BYERS, M. D.,
Charlotte, N. C.
—*Medical Record.*

